# Supplementary material for: P-TRAP: a Panicle Trait Phenotyping tool
Source: BMC Plant Biol. 2013 Aug 29;13:122. doi: 10.1186/1471-2229-13-122 (PMC3848748; doi:10.1186/1471-2229-13-122)
Supplement: Additional file 7 — P-TRAP source code. The Netbeans project that contains the source code of the application. [file 1471-2229-13-122-S7.zip › javadoc.zip!/javadoc/org-bioalg-imageprocessor/org/bioalg/imageprocessor/package-summary.html]

org.bioalg.imageprocessor (Image Processor)


JavaScript is disabled on your browser.


- Package
- Class
- Tree
- Deprecated
- Index
- Help

*org.bioalg.imageprocessor 1.0*

- Prev Package
- Next Package

- Frames
- No Frames

- All Classes

# Package org.bioalg.imageprocessor

- Class Summary

  | Class | Description |
  | --- | --- |
  |  |  |
  | --- | --- |
  | AImage | The main class used for image processing of the project. |
  | ColoredImage | The colored image class. |
  | EDistanceMap | The distance mapper of the image. |
  | HSBPixel | The Hue Saturation Brightness model. |
  | ImageIO | The Input Output operations of the image based on both generic java imaging and JAI imageing. |
  | Kernels | Several kernels created by this class. |
  | MathMorphology | The Mathematical morphology handler. |
  | RGBPixel | The Red Green Blue model of the pixel. |
- Enum Summary

  | Enum | Description |
  | --- | --- |
  |  |  |
  | --- | --- |
  | AImage.CONNECTIVITY |  |
  | MathMorphology.MorphingType |  |

- Package
- Class
- Tree
- Deprecated
- Index
- Help

*org.bioalg.imageprocessor 1.0*

- Prev Package
- Next Package

- Frames
- No Frames

- All Classes
